# Supplementary figures and images for: Yak rumen microbial diversity at different forage growth stages of an alpine meadow on the Qinghai-Tibet Plateau
Source: PeerJ. 2019 Sep 19;7:e7645. doi: 10.7717/peerj.7645 (PMC6754979; doi:10.7717/peerj.7645)

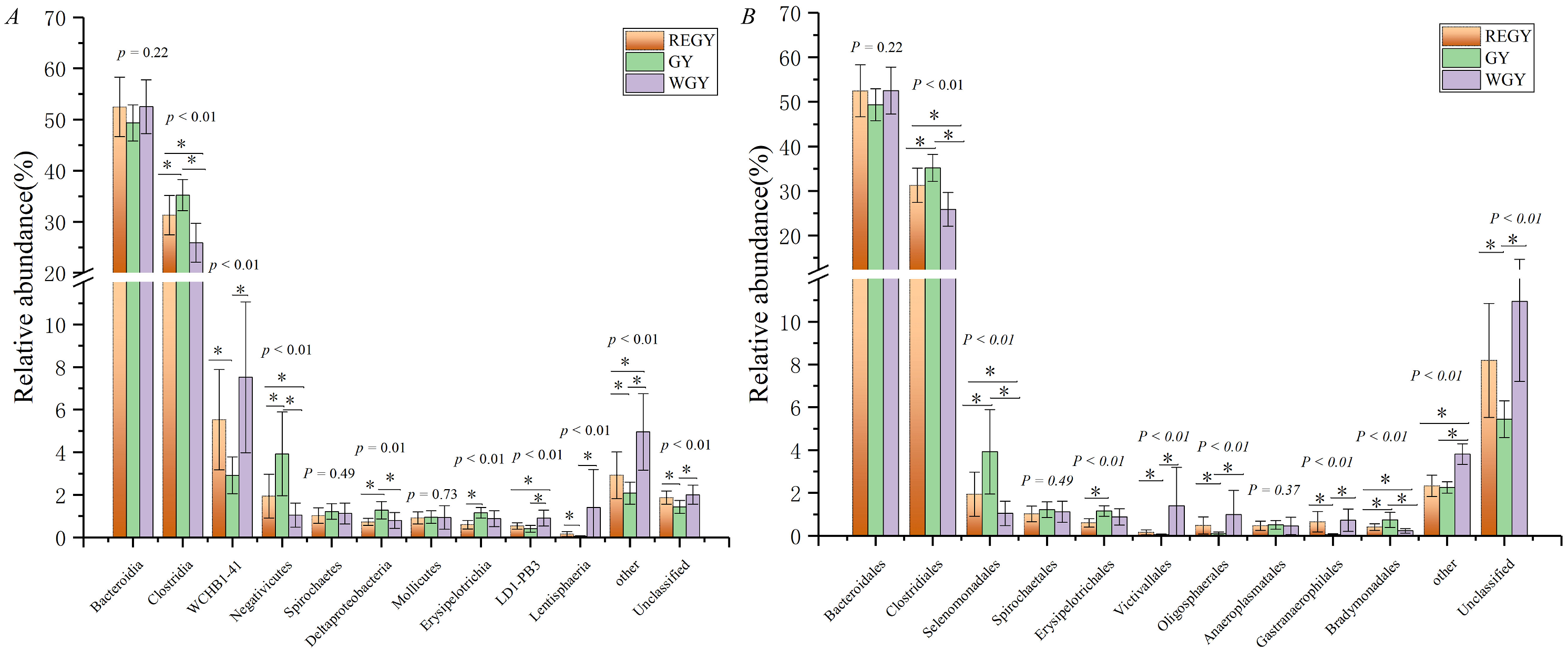

Supplement: Figure S1 — (A) The abundance of the top 10 Class at different grass growth stages. (B) The abundance of the top 10 Order at different grass growth stages. The significant difference in groups by Kruskal–Wallis test with p < 0.05 marked, the extremely significant difference in groups by Kruskal–Wallis test with p < 0.01 marked. The one-asterisk Above the column represents that there is significant difference in between groups (P value <0.01). [file peerj-07-7645-s001.png]

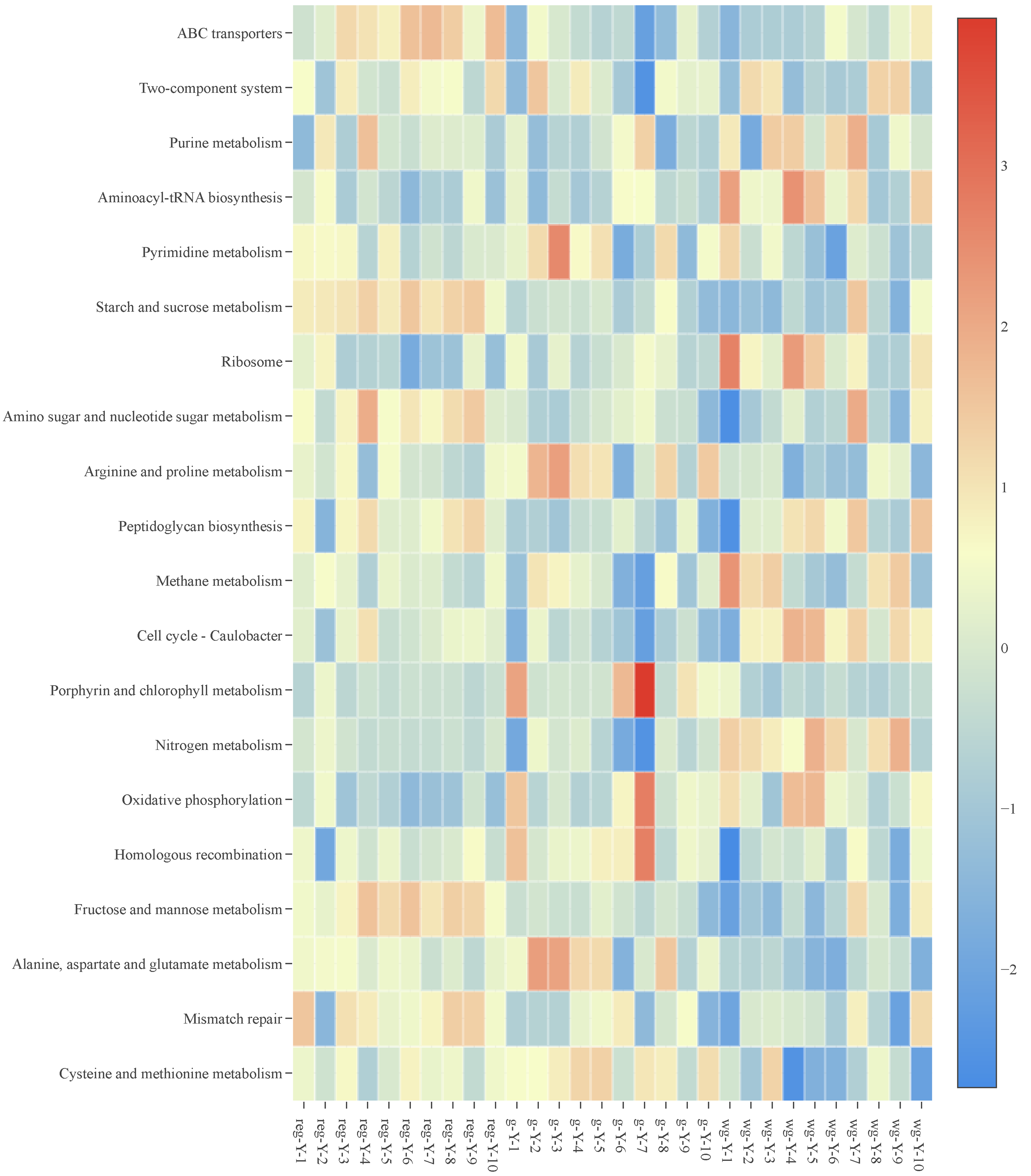

Supplement: Figure S2 — The different color represented the relative abundance of KOs in KEGG level 3 pathway. [file peerj-07-7645-s002.png]
